# RNA sequencing and proteomic profiling reveal alterations by MPTP in chronic stomach mucosal injury in tree shrew Chinese (*Tupaia belangeri chinensis*)

**DOI:** 10.1038/s41598-023-50820-y

**Published:** 2024-01-02

**Authors:** Chen-yun Wang, You-song Ye, Wei-hu Long, Zhe-li Li, Hong Zheng, Xiao-rui Lin, Wei Zhou, Dong-hong Tang

**Affiliations:** 1 Medical Primate Research Center of China, Institute of Medical Biology, Chinese Academy of Medical Sciences/Peking Union Medical College, Kunming, 650118 China; 2 Kunming Medical University, 1168 West Chunrong Road, Yuhua Avenue, Chenggong District, Kunming, 650504 Yunnan People’s Republic of China

**Keywords:** Molecular biology, Neuroscience, Gastroenterology

## Abstract

1-Methyl-4-phenyl-1,2,3,6-tetrahydropyridine (MPTP) is a neurotoxin that can cause gastrointestinal ulcers by affecting dopamine levels. Therefore, MPTP has been considered a toxic substance that causes gastric ulcer disease in experimental animals. In this study, tree shrews were used as the animal model of gastric mucosa injury, and MPTP was intraperitoneally injected at a lower MPTP dosage 2 mg/kg/day for 13 weeks, while tree shrews were not injected as the control group. Under the light microscope, local congestion or diffuse bleeding points of gastric mucosa and multiple redness and swelling bleeding symptoms on the inner wall were observed in the treatment group, as well as immune cell infiltration was found in HE staining, but no such phenomenon was observed in the control group. In order to explore the molecular basis of changes in MPTP induced gastric mucosa injury, the transcriptome and proteome data of gastric mucosa were analyzed. We observed significant differences in mRNA and protein expression levels under the influence of MPTP. The changes in mRNA and proteins are related to increased immune infiltration, cellular processes and angiogenesis. More differentially expressed genes play a role in immune function, especially the candidate genes *RPL4* and *ANXA1* with significant signal and core role. There are also differentially expressed genes that play a role in mucosal injury and shedding, especially candidate genes *GAST* and *DDC* with certain signaling and corresponding functions. Understanding the factors and molecular basis that affect the expression of related genes is crucial for coping with Emotionality gastric mucosa injury disease and developing new treatment methods to establish the ability to resist disease.

## Introduction

1-Methyl-4-phenyl-1,2,3,6-tetrahydropyridine (MPTP) is a potent neurotoxin used to selectively destroy dopaminergic neurons in the substantia nigra and induce parkinsonism. MPTP is metabolised to the 1-methyl-4-phenylpyridinium ion (MPP+) in glia, after which it enters the neuron via the dopamine transporter and results in elevated levels of oxidative stress^1^. MPTP, as a neurotoxin, causes a decrease in dopamine content in the endocrine system of animals by disrupting dopaminergic neurons in the substantia nigra of the midbrain. Dopamine is an important gastrointestinal neurotransmitter derived from tyrosine, which participates in the regulation of human emotions (feelings, desires), and participates in the occurrence of gastrointestinal peptic ulcer by combining with gastrointestinal dopamine receptors (DR). MPTP has been considered as a toxic agent causing ulcer disease in experimental animals. Intraperitoneal injection of MPTP can cause gastric and duodenal mucosa damage or ulcers in rats^2,3^. In addition, it had found that intraperitoneal injection of MPTP can cause significant damage to the gastric and duodenal mucosa of rats, manifested as mucosal erosion and ulcer, and the damage index is 5–6 times higher than that of the control group^4^.

Gastrointestinal diseases caused by gastrointestinal mucosal injury are a common acute and chronic digestive system diseases in clinical practice, which often occur repeatedly and bring great harm to the physical and mental health of patients. Establishing an ideal animal model of gastrointestinal mucosal injury in the digestive tract is of positive significance for exploring the etiology, pathogenesis, progression of the disease, clarifying treatment methods, and developing new drugs. The genetic research of tree shrew (*Tupaia belangeri*) shows that it is closer to primates than rodents in biology, and has a significantly larger brain and higher nervous system than rats. The application of tree shrews in the research of related diseases has a good prospect.

Previous studies found that a few lymphocytes and plasma cell infiltrated the gastric mucosa of tree shrews after intraperitoneal injection of MPTP, (the dosage of MPTP was 2 mg/kg, i.p) and part of the surface mucosa fell off. A few tree shrews can see obvious damage to gastric mucosa, which is highlighted by submucosal cell edema, structural disorder of some gastric mucosa, accompanied by bleeding and shedding of necrotic mucosa, or mucosal epithelium shedding, lamina propria congestion, and inflammatory cell infiltration, goblet cell increase, submucosal congestion and edema with inflammatory cell exudation, and a large number of inflammatory cell infiltration between muscle fibers^5^. However the molecular basis of this lesion is not yet understood.

In this study, tree shrews were used as animal models, and long-term low-dose intraperitoneal injection of MPTP was used to establish a model of gastrointestinal mucosal injury in tree shrews. By combining transcriptome and proteome, we explored the key pathway of MPTP induced gastrointestinal mucosal damage in tree shrews and the molecular basis of differentially expressed proteins.

## Results

### Physiological parameters of stomach mucosal injury

Morphological observation showed that the size and color of the stomachs of T group were normal. However, the surface of the stomachs was observed to be full of bruises and the contents were black (Supplementary Fig. 1A and B). Under stereopicroscope, the samples all showed different degrees of gastric mucosa injury, which was specifically manifested as local congestion or diffuse hemorrhagic spots on the gastric mucosa, multiple redness and bleeding on the inner wall covered with white moss, or partial slight shedding of the gastric mucosa, many places showed strange pink, and zero scattered on the inner wall of slight hemorrhagic spots (Supplementary Fig. 1C and D). In the control group, there were no abnormal gastric manifestations, little stomach contents, complete and clean gastrointestinal mucosa, complete structure, normal size, shape and color (Supplementary Fig. 1). Besides, a few lymphocyte and plasma cell infiltrates can be seen in gastric mucosa (1–10%) (Supplementary Fig. 1E and F).

### Principal component and hierarchical cluster analyses

We quantified mRNA by RNA-Seq and detected expression of 22,271 genes.

**Figure 1:**
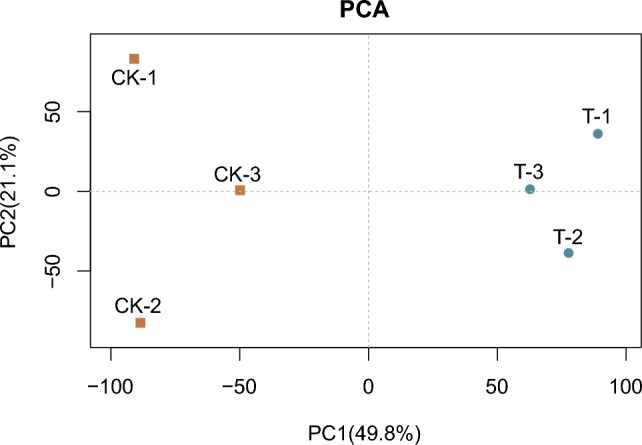
PCA of RNA transcripts (n = 3). Red represents the control group CK and blue represents the administration group T.

PCA revealed a clear separation between the two groups (CK and T) indicating distinct RNA expression patterns (Fig. 1).

### Mucosal injury changes in mRNA expression

**Figure 2:**
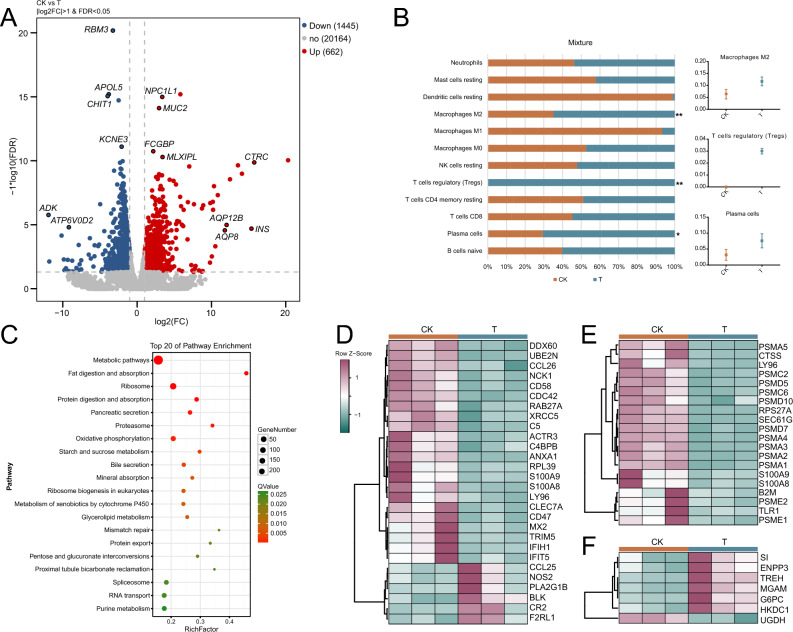
Analysis of differentially expressed mRNA. (**A**) Volcano plots of total RNA-sequencing showing DEGs between two groups. Some of the genes with the most significant differences are highlighted. (**B**) Abundance of immunoinfiltrating cells inferred from transcripts. The box diagrams on the right show immune cell types with significant differences in abundance (*p < 0.05, **p < 0.01). Red represents the control group CK and blue represents the administration group T. (**C**) Bubble plots of KEGG enrichment analysis for the significantly DEGs set with top 20 of pathway enrichment. (**D**) The heatmap of 28 genes related to innate immune response between two groups. (**E**) The heatmap of 20 genes related to antigen processing-Cross presentation between two groups. (**F**) The heatmap of 7 genes related to starch and sucrose metabolism between two groups.

Comparing the T group with the CK group revealed 2107 differentially expressed genes (DEGs) (662 up/1445 down), including some genes with most significantly differentiated signals (*RBM3*, *APOL5*, *CHIT1*, *KCNE3*, *ADK*, *ATP6V0D2*, *NPC1L1*, *MUC2*, *FCGBP*, *MLXIPL*, *CTRC*, *AQP12B*, *INS*, *AQP8*)(Fig. 2A). Some genes are involved in functions related to gastric mucosal damage and histopathologic change. Adenosine kinase (ADK) is critical for neointima formation after vascular injury by inducing aberrant DNA hypermethylation^6^. ATP6V0D2 can mediate leucine-induced mTORC1 activation in macrophages, which further regulates macrophage differentiation^7^. MUC2 (Mucin 2), which is produced by the goblet cells, forms the skeleton of the intestinal mucus and protects the intestinal tract from self‐digestion and numerous microorganisms^8^.

In the enrichment results of differentially expressed genes, we actually found many immune-related terms and pathways which was consistent with the pathological changes of gastric mucosa (such as the appearance of lymphocytes and plasma cell infiltration). However, in immunoinfiltration analysis using transcript expression information, it was indeed found that the abundance of macrophages M2, T cells regulatory and plasma cells in the T group was significantly higher (*p* < 0.05) than that in the CK group (Fig. 2B).

Gastric mucosal injury is accompanied by immune system changes such as the appearance of lymphocytes and plasma cell infiltration, as well as surface mucosal shedding, bleeding, goblet cell proliferation and other phenomena. Interestingly, in the GO enrichment results, some differential expression genes were significantly enriched in terms of immunity, cellular processes, metabolism, response to stimuli, membrane and biological regulation (e.g., GO:0,002,376, GO:0,050,896, GO:0,065,007, GO:0,009,987, GO:0,016,020 etc.) (Supplementary Table 1). Notably, KEGG analyses revealed that many genes of differential expression are enriched in pathway of gastrointestinal function, e.g., Metabolic pathways, proteasome, digestion and absorption of lipids and protein, secretion of pancreatic fluid and biliary, mineral absorption, purine metabolism etc.(Fig. 2C). In addition, there are also some immune-related pathways, such as antigen processing and presentation, intestinal immune network for IgA production and RIG-I-like receptor signaling pathway etc. (Supplementary Table 2). These differentially expressed genes may be significantly correlated with gastric mucosal injury.

The heat map of selected 55 DEGs associated with innate immune response, antigen processing-Cross presentation, starch and sucrose metabolism showed significant differences between the two groups (Fig. 2D–F). The changes of immune system related genes and the metabolism of starch and sucrose may be directly related to the damage of gastric mucosa and the influence of gastric function.

**Figure 3:**
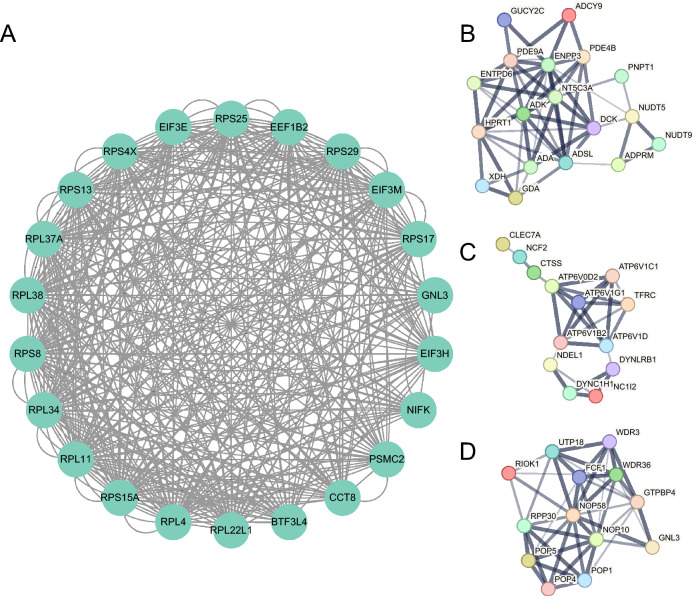
Network analysis with differentially expressed genes. (**A**)The subnetwork with highest degree estimated by MCODE in Cytoscape. Network analysis show overrepresentation of genes involved in purine metabolism (**B**), phagosome (**C**) and ribosome biogenesis in eukaryotes (D).

The DEGs was analyzed by the online tool STRING and Cytoscape software shown in Fig. 3. Some genes in subnetwork with the highest degree score in Cytoscape may be closely related to mucosal injury (Fig. 3A). In total, 22 nodes and 388 edges were identified by the MCODE tool in the Cytoscape software. *EIF3E* was found which is an angiogenic inhibitor that promotes angiogenesis after ischemic injury. Silencing of *EIF3E* promotes blood perfusion recovery after limb ischemia through stabilization of hypoxia-inducible factor 2α activity^9^. And research has also found that Int6/ *EIF3E* silencing promotes functional blood vessel outgrowth and enhances wound healing by upregulating hypoxia-induced factor 2alpha expression^10^. Some genes may be involved in gastric mucosal damage and abscission, for example, the results of a study suggest that RPL34 plays a critical role in cell proliferation, cell cycle distribution and apoptosis of human malignant gastric cells^11^. Besides, among the results, 3 clusters which may be involved in the functions of purine metabolism, phagosome and ribosome biogenesis in eukaryotes and is likely the result of immune cell infiltration into the gastric mucosa. We found that some core genes contained in the networks, including some of the genes (*ADK* and *ATP6V0D2*) with the most significant signals mentioned above, as well as some other core genes (e.g., *ADA*, *HPRT1*, *NT5C3A*, *DCK*, *ADSL*, *DYNC1H1*, *ENPP3*, *PDE9A*, *ENTPD6*, *FCF1*, *NOP58*, *WDR3*, *WDR36*, *GTPBP4*, *NOP10*, *POP4* etc.), which play key roles in related functional networks (Fig. 3B–D). Hypoxanthine–guanine phosphoribosyltransferase (HGPRT, also known as HPRT1) is a key enzyme in the purine salvage pathway^12^. *DYNC1H1* loss of function caused a significant decrease in cell viability and cell proliferative ability, inhibition of the cell cycle^13^. In addition, the study found that depletion of *NOP10* promotes oxidative stress and disrupts ribosomal biogenesis^14^.

Together these gastric mucosal injury related changes in transcripts indicate infiltration of immune cells, cell viability, and cell proliferative ability etc.

### Mucosal injury changes in protein expression

**Figure 4:**
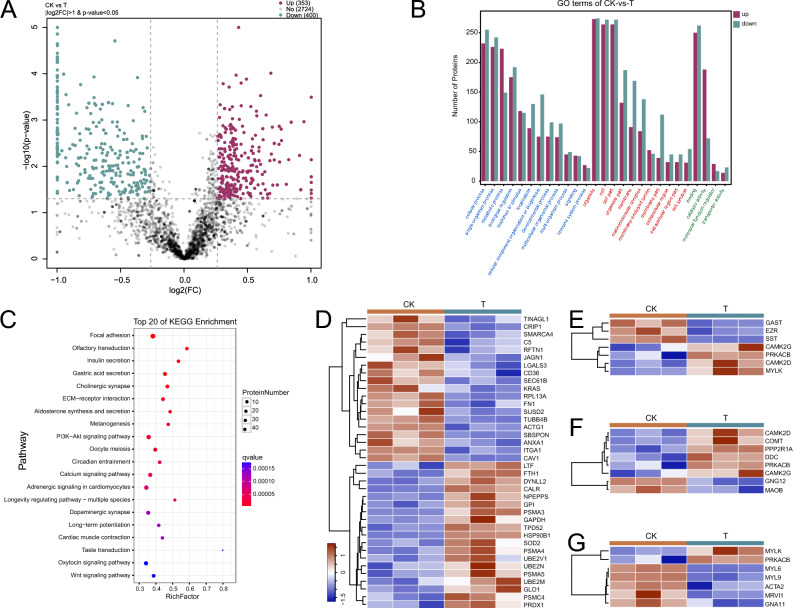
Analysis of differentially protein expression. (**A**) Volcano plots of total expressed protein showing differentially expressed proteins between two groups. (**B**) Functional enrichment of differentially expressed proteins with histogram of GO enrichment analysis, which observed overrepresentation of genes associated with terms (adjusted *p*-value < 0.01) representing a wide array of biological processes (blue font), cellular compartments (red font) and molecular functions (green font). (**C**) Bubble plots of KEGG enrichment analysis for the significantly DEPs set with top 20 of pathway enrichment. (**D**) The heatmap of 38 differentially expressed proteins related to immune system process between two groups. (**E**) The heatmap of 7 differentially expressed proteins related to gastric acid secretion between two groups. (**F**) The heatmap of 8 differentially expressed proteins related to dopaminergic synapse between two groups. (**G**) The heatmap of 7 differentially expressed proteins related to vascular smooth muscle contraction between two groups.

Comparing the T group with the CK group revealed 753 (353 up/400 down) differential expression proteins (DEPs) (Fig. 4A). Proteins (ADA, ALDOB, ASS1, BGN, CA3, CALB2, COL1A2, COL6A1, COL6A2, CRIP1, FABP1, FTH1, HSP90B1, MYH10, MYH11, MYL6, OGN, PRSS2, RBP2, RPL18, SERPINA1, SERPINA6, SERPINC1, SOD1, SST, VASP, XPOT etc.) with significant differential expression signals may play an important role in gastric mucosa injury. The adenosine deaminase (ADA) activity was highest in the stomach of mice on normal diet^15^. ADA possess a crucial role of this enzyme in the control of vascular inflammation^16^ and promoting autoreactive T cell activation^17^. Autoantigen carbonic anhydrase III (CA3), which detected in serum lexes of patients with hyperplasia, has a corresponding relationship with microscopic polyangiitis (MPA)^18^. It means that CA3 may have a certain relationship with submucosal congestion in gastric mucosal injury. Overexpression of CRIP1 in transgenic mice alters cytokine patterns and the immune response^19^. Studies have found that loss of osteoglycin (OGN) promotes angiogenesis in mouse models via modulation of vascular endothelial growth factor and vascular endothelial growth factor receptor 2 signalling pathway^20^. In other words, the protein has a negative correlation with angiogenesis, and the down-regulation of the protein expression may be related to the vascular damage and repair phenomenon corresponding to the hyperemia in the lower layer of gastric mucosa after injury. In the study of macrophage anti-inflammatory properties found that the regulation of SERPINA1 contributes to the anti-inflammatory and host defense properties^21^. When gastric mucosa is inflamed and damaged, the physiological response may cause the protein expression to be up regulated.

We observed many consistent terms and pathways between protein enrichment results and transcriptome differential expression genes enrichment results. Gene Ontology (GO) analyses revealed that many proteins of differential expression are also enriched in terms of immunity, cellular processes, metabolic process, response to stimulus, membrane and biological regulation (e.g., GO:0002376, GO:0050896, GO:0065007, GO:0009987, GO:0016020 etc.) (Fig. 4B and Supplementary Table 3). KEGG analyses revealed that many genes of differential expression are enriched in pathway of gastrointestinal function and key neurotransmitter, e.g., insulin secretion, gastric acid secretion, PI3K − Akt signaling pathway, dopaminergic synapse etc. (Fig. 4C and Supplementary Table 4). These differentially expressed proteins may be significantly correlated with gastric mucosal injury.

The heat map of selected 60 DEPs associated with immune system process, gastric acid secretion, dopaminergic synapse, vascular smooth muscle contraction showed significant differences between the two groups (Fig. 4D–G). The changes of proteins related immune system, gastric acid, dopaminergic synapse and vascular may be directly related to the damage of gastric mucosa and the influence of gastric function.

### Integration of transcriptomic and proteomic analyses, comparing MPTP group and control group

**Figure 5:**
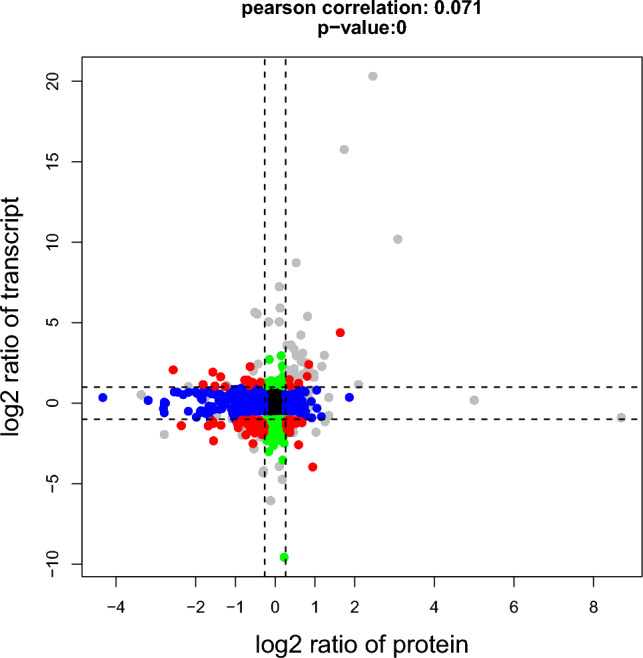
Comparison of gastric mucosa injury related changes in mRNA and protein. We identified 2481 genes significant differences for both mRNA and protein. These can be divided in four groups depending on the direction of change (red), with decreased RNA and increased protein (bottom right), decreased RNA and decreased protein (bottom left), increased RNA and increased protein (top right), and increased RNA and decreased protein (top left).

In order to compare gastric mucosal injury related trends in mRNA and proteins, We identified genes with both mRNA and protein data of which 2481 had significant differences. These can be divided in four groups depending on the direction of change, with decreased RNA and increased protein (46 genes), decreased RNA and decreased protein (46 genes), increased RNA and increased protein (9 genes), and increased RNA and decreased protein (16 genes) (Fig. 5). The broad range of non-overlapping features and the consequent identification of several non-overlapping pathways between the mRNA and protein could indicate certain selective effects from MPTP on protein and RNA expression in the stomach mucosa. There were 55 genes with consistent changes, accounting for 2.22%. There are a lot of functional genes involved PRSS2, ACAA2, ADA, ALDOB, ANXA1, ATP5J, C5, CDH17, DDC, DYNC1H1, EEF1A1, GAST, GKN1, GKN2, HNRNPH2, MRPS36, NDUFA12, RPL24, RPL26, RPL27, RPL34, RPL4, RPS23, RPS29, RPS6, RPS8, SNAP23, SRSFc1, TPM4, USMG5, VDAC2. Some gene functions are closely related to gastric mucosal injury in this study, such as: RPL4 acts in B lymphoblasts which was been found EBV uses NCL and RPL4 to establish persistent B-lymphoblastoid cell infection^22^. Knockdown of Annexin A1 (ANXA1) induces apoptosis, causing G2/M arrest and facilitating phagocytosis activity in human leukemia cell lines^23^. Gastrin (GAST) induces parathyroid hormone-like hormone expression in gastric parietal cells, which might contribute to gastric epithelial cell homeostasis^24^. L-Dopa Decarboxylase (DDC) is a pyridoxal requiring enzyme that catalyzes the decarboxylation of L-3,4-dihydroxyphenylalanine (L-Dopa) to Dopamine (DA). A previous study support the involvement of DDC in apoptosis, reflect the great importance of the DA synthesizing enzyme in important physiological processes as well as in pathological conditions ranging from cancer to neurodegenerative diseases^25^. Besides, These 55 genes were significantly enriched in terms of purine nucleoside triphosphate metabolic process (GO:0009144), immune effector process (GO:0002252) and regulation of apoptotic signaling pathway (GO:2001233). ADA gene, as a key gene in the above immune process, reappeared, indicating that there was significant consistency between mRNA transcription and protein expression of this gene in gastric mucosal injury. In addition to affecting the cell cycle, DYNC1H1 regulates cell growth and metastasis by IFN-γ-JAK-STAT signaling and is associated with an aberrant immune response^13^. The above two genes are also the key genes in the results of the gene interaction network (Fig. 3), which plays an important role in the immune process or immune cells. However, Some genes are uniformly down-regulated in both mRNA and protein expression, such as ANXA1, C5 etc. In the appeal, Knockdown of Annexin A1 (ANXA1) induces apoptosis, causing G2/M arrest and facilitating phagocytosis activity in human leukemia cell lines^23^. Production of autoantibodies, T cell activation, and T cell cytokine production were not affected by the absence of C5 and thrombin activates C5 to provoke arthritis^26^. Similarly, in the process of immune response caused by gastric mucosa injury, the expression and translation of some genes will promote immune response through downregulation.

We identified 62 genes with inconsistent RNA and protein expression, including decreased RNA and increased protein (46 genes), increased RNA and decreased protein (16 genes). These genes were significantly enriched in terms of regulation of cellular response to stress (GO:0080135), cellular detoxification (GO:1990748), leukocyte migration (GO:0050900) and regulation of protein kinase activity (GO:0045859). It also contains some functional genes, for example, the study of cell activity revealed a new regulatory mechanism of HSPB6 in cell survival through its interaction with BECN1^27^. In a study of lung inflammation found PPIA can affect the foaming of macrophages and may participate in silicosis fibrosis^28^. It is interesting to note that for these post-transcriptional processes, the direction of change differs between mRNA and proteins, thus hinting at possible gastric mucosal injury related by MPTP post-transcriptional regulation mechanisms at play.

### Relating mRNA and protein changes to physiology in stomach mucosal injury

**Figure 6:**
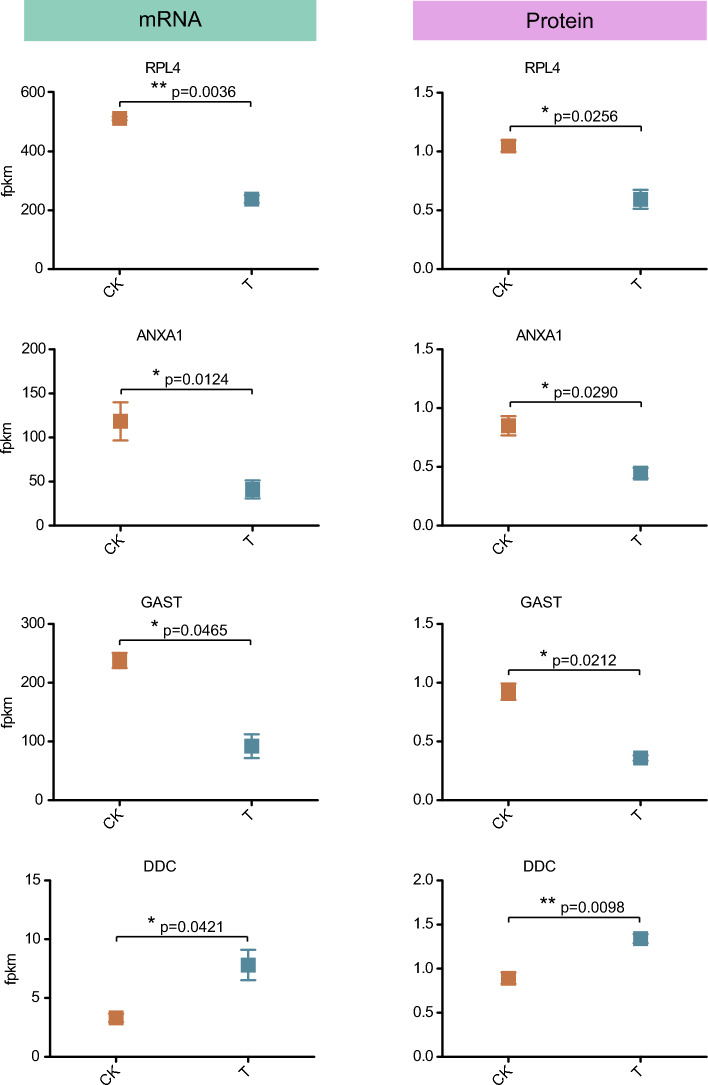
Examples of 4 genes with consistent direction of gastric mucosa injury related changes between mRNA and protein. Red represents the control group CK and blue represents the administration group T.

Gastric mucosal injury is associated with cell apoptosis and membrane homeostasis. In addition, the presence of lymphocytes, plasma cells and other immune infiltrations accompanied by gastric mucosal injury, as well as partial submembrane edema and mucosal necrosis and shedding, mean more activation of the immune system. This phenomenon was indeed consistent in the immunoinfiltration analysis, namely, the abundance of macrophage M2, T cell regulatory and plasma cells increased significantly. The 6 genes (*RPL4*, *ANXA1*, *GAST*, *DDC*, *ADA*, *DYNC1H1*) with the same direction of mRNA and protein changes associated with gastric mucosa injury did have significant differences between the groups (Fig. 6 and Supplementary Fig. 2). The results showed that the expression levels of gene *DDC* (mRNA and protein) in the T group were significantly upregulated compared to the CK group, while other genes (*RPL4*, *ANXA1*, *GAST*, *ADA*, *DYNC1H1*) were significantly downregulated. The mechanism of some changes in cell homeostasis and immune infiltration associated with gastric mucosa injury may be closely related to changes in the expression of related genes.

**Figure 7:**
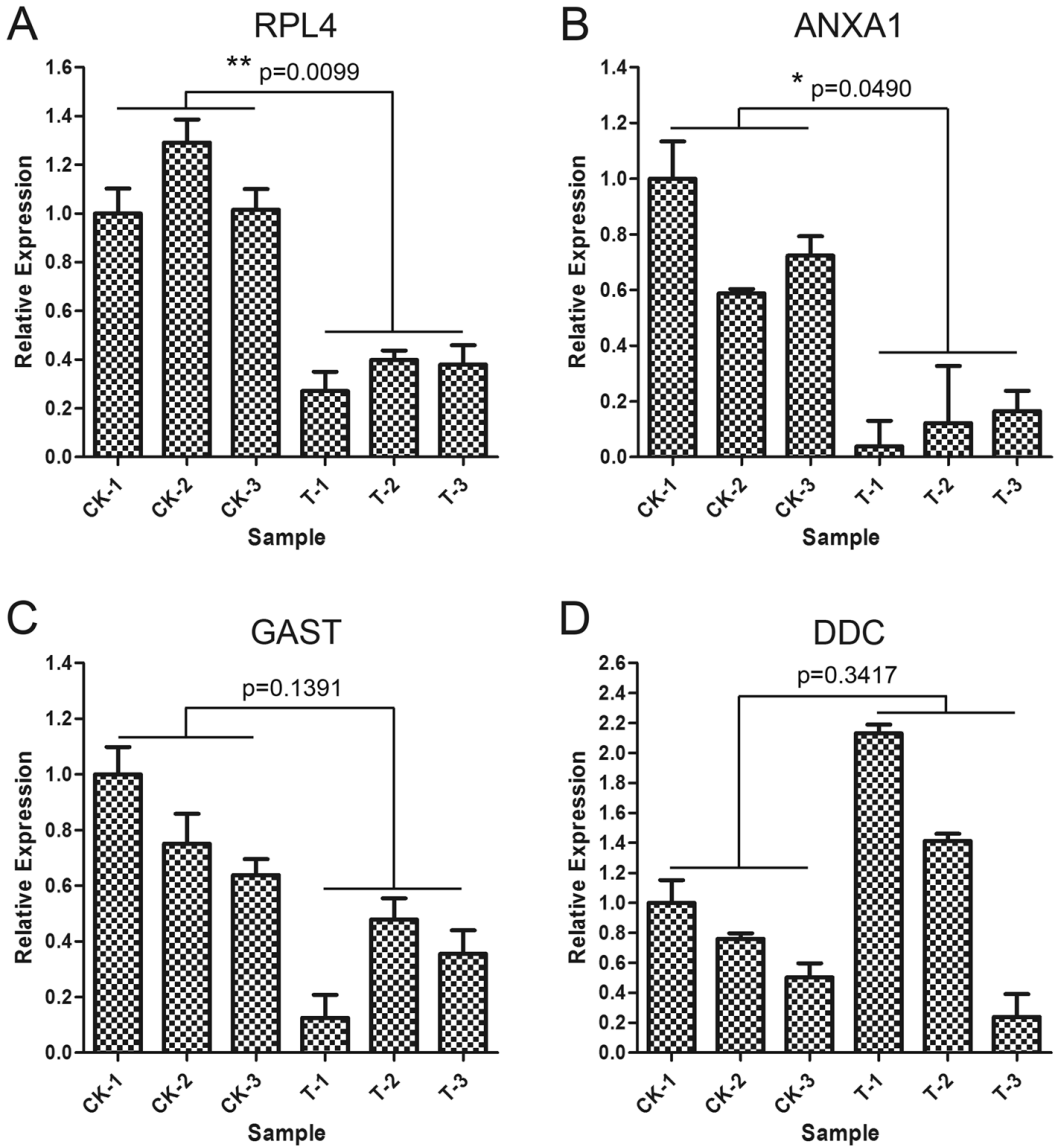
The real-timePCR experiment of 4 genes with consistent direction of gastric mucosa injury related changes between mRNA and protein.

In addition, we conducted real-timePCR experiments to verify the above four genes (*RPL4*, *ANXA1*, *GAST, DDC*), and obtained consistent results (Fig. 7), which also demonstrated the effectiveness of the candidate genes. But the results showed significant differences in the expression of two genes (*RPL4*, *ANXA1*), while the expression of the other two genes (*GAST, DDC*) was not significant.

## Discussion

Gastrointestinal diseases are common and frequently occurring, with common mucosal damage as the main lesion. Psychological factors and unhealthy lifestyle habits can all be induced. With the accelerated pace of life, bad living habits, and increased social, work, family, and psychological burdens, the incidence rate of the disease has increased. MPTP is a neurotoxin that selectively disrupts the dopamine content of dopaminergic neurons in the substantia nigra of the midbrain. This will affect people's emotions, and finally, the binding of dopamine receptors in the gastrointestinal tract will be affected, leading to the occurrence of gastrointestinal peptic ulcer.

In this study, MPTP drug injection was carried out in the established animal model (tree shrew), and gastric mucosa injury symptoms were indeed found in T group. In this study, using transcriptomic and proteomic analyses of stomach tissue from tree shrews, we observed that effects of MPTP in stomach mucosal injury by differential expression of transcriptomic and proteomic. MPTP has been frequently used in Parkinson's disease research in the past, as a neurotoxin, and is often used in the preparation of animal models of Parkinson's disease^29^. But it has also been found to cause gastrointestinal lesions, the molecular basis of which is not yet clear.

Our previous studies found long-term intraperitoneal injection with a low dose of MPTP is a feasible method for the establishment of a tree shrew model of chronic gastrointestinal mucosal injury. The optimal dose is 2 mg/kg daily for at least 56 consecutive days. In this study, we didn’t observed that tree shrews died in this MPTP-created chronic model at the dosage of 2 mg/kg/day (i.p) MPTP during 13 weeks. We has previously also observed the mortality after MPTP administration in four dose groups^30^. It had been shown that after injection with an MPTP dosage of 20 mg/kg, all 10 of the tree shrews died after 1 day. After injection with 10 mg/kg of MPTP, eight tree shrews died after 1 day, and the remaining two tree shrews died within three days. Only one tree shrew survived for 4 days after injection with 5 mg/kg MPTP, and it died on the fifth day. When the dosage of MPTP was 3 mg/kg, no tree shrews died until the fifth day.

The present study screened the transcripts and proteins in gastric mucosa damage and non-damage of tree shrews. To clarify the functions of these differentially expressed mRNAs and proteins, we conducted GO enrichment and KEGG pathway analysis, as well as PPI network analysis. The enrichment and pathway analysis indicated these differentially expressed RNAs and proteins were involved in biological regulation, cellular component organization or biogenesis, metabolism, metabolic pathways, oxidative phosphorylation and so on. In the gastric mucosal injury, the following functions may play critical roles: immunity, cellular processes, response to stimuli, membrane and biological regulation, digestion and absorption of lipids and protein, mineral absorption, purine metabolism, gastric acid secretion, PI3K–Akt signaling pathway, dopaminergic synapse etc. These results indicated that the immune response, cellular processes, metabolisms are major functional alterations in gastric mucosal injury. In addition, it has been found that some genes play important roles in cell cycle, immune cells, macrophages, angiogenesis, and other processes, which were consistent with our GO and KEGG analysis.

To further screen out the key genes which may play critical roles in the gastric mucosal injury, we analyzed the consistency of transcriptome and proteome in the present study. Surprisingly, only a few genes were screened out. Some genes, such as RPL4, ANXA1, GAST, C5 etc. were downregulated in gastric mucosal injury at both mRNA and protein levels. Some genes, such as *PRSS2*, *DYNC1H1*, *ADA*, *ALDOB*, *CDH17* and *DDC*, were upregulated in gastric mucosal injury at both mRNA and protein levels. ADA possess a crucial role of this enzyme in the control of vascular inflammation^16^ and promoting autoreactive T cell activation^17^. DYNC1H1 regulates cell growth and metastasis by IFN-γ-JAK-STAT signaling and is associated with an aberrant immune response^13^. Combined with the immune infiltration, cell edema and necrosis, and the increase of goblet cell associated with the tree shrew gastric mucosa injury, it shows that the above two candidate genes play critical roles in the gastric mucosa injury induced by MPTP. However, the functions of these two genes need to be verified by further functional experiments.

However, we found that some consistently expressed genes above play important roles in immune function or gastric mucosa injury and abscission, especially the candidate genes (*RPL4*, *ANXA1*, *GAST, DDC*) with significant signal and core role in results of omics data analysis and functional experiment. *RPL4* knockdown decreased EBNA1 activation of an oriP luciferase reporter, EBNA1 DNA binding in lymphoblastoid cell lines, and EBV genome number per lymphoblastoid cell line^22^. And knockdown of Annexin A1 (ANXA1) induces apoptosis, causing G2/M arrest and facilitating phagocytosis activity in human leukemia cell lines^23^. In other words, down-regulated expression of *RPL4* and *ANXA1* gene may contribute to the function of immune cells. Gastrin (GAST) induces parathyroid hormone-like hormone expression in gastric parietal cells, which might contribute to gastric epithelial cell homeostasis^24^. L-Dopa Decarboxylase (DDC) is a pyridoxal requiring enzyme that catalyzes the decarboxylation of L-3,4-dihydroxyphenylalanine (L-Dopa) to Dopamine (DA), which was supported the involvement of DDC in apoptosis^25^. The down-regulation of *GAST* gene and up-regulation of *DDC* gene may more directly cause the mucosal cell death and abscission associated with gastric mucosal injury. Although the expression difference of these two genes (*GAST* and *DDC*) was not significant in RT-PCR results (Fig. 7), the expression difference trend was consistent with the transcriptome and proteome (Fig. 6). If the number of repeated experiments is increased, significantly different results may occur. Understanding the factors and molecular basis that affect the expression of related genes will be crucial for coping with Emotionality gastric mucosa injury disease and developing new treatment methods to establish the ability to resist disease.

In the transcriptome and proteome differential expression information, we found that the expression of some genes and proteins was not consistent with the up-regulation and down-regulation, which may be involved by RNA splicing^31^. The lncRNAs and cirRNAs are involved in the gene regulation by many methods, such as binding to target genes, affecting the histone modification, activating transcriptional factors, and binding to miRNA as competitive endogenous RNA (ceRNA). Whether these have an effective effect on the differences between the transcriptome and proteome in gastric mucosal injury needs to be further studied in the future.

As a neurotoxin, MPTP affects some neurological disorders in tree shrews and indirectly damages gastric mucosa through dopamine. This pathological injury may stimulate the corresponding immune response and damage repair function in tree shrews. Therefore, more functions of the two groups of differentially expressed genes were concentrated in immunity, cellular processes and angiogenesis. Our study shows that there are significant differences in the transcription and expression of genes caused by gastric mucosal injury. To understand the mechanism of how dopamine, as a gastrointestinal neurotransmitter, affects gastric mucosal injury, more tissue and data information are needed to carry out research.

## Materials and methods

### Experimental animals and ethics statement

Six 2–3-year-old adult male and female Chinese tree shrews (subfamily *Tupaia belangeri Chinensis*) were used in this study, with body weights ranging from 110 to 140 g. They were obtained from the colony of the Medical Primate Research Center of the Institute of Medical Biology, Chinese Academy of Medical Science, and Peking Union Medical College (Kunming, China).

Animal welfare and experimental procedures were conducted in accordance with the Guide for the Care and Use of Laboratory Animals (National Research Council Institute for Laboratory Animals, R. (1996), Washington (DC), National Academy Press (US)). All efforts were made to minimize animal suffering. The Institutional Animal Care and Welfare Committee of the Institute of Medical Biology, Chinese Academy of Medical Sciences, and Peking Union Medical College approved this study, and all procedures were performed according to ethical standards and practices. All procedures, including animal care and tissue collection protocols, were conducted in accordance with ARRIVE guidelines and Institutional Animal Care and Use regulations and rules (Approval No.: DWSP2017058).

### Drug treatment

MPTP was purchased from Sigma-aldrich Corporation Co.. MPTP was dissolved in normal saline to a concentration of 1 mg/mL for the treatment of animal. Six tree shrews were divided into two groups of three. One group was given daily intraperitoneal administration (MPTP) with 2 mg/kg/day for 13 weeks, defined as experimental group T, and the other group was not treated, defined as control group CK.

### Tissue sampling

After 13 weeks, the tree shrew was euthanized through dislocation. Then we perform a laparotomy and remove the entire stomach from the pylorus and cardia. Subsequently, we use a double-sided blade to open the stomach along the greater curvature of the stomach, and use a syringe to draw Bouin solution to rinse the stomach, then wipe off the contents. Observe the gastric mucosa using a stereomicroscope and take photos for recording. Afterwards, the gastric body was taken and continued to be fixed in Bouin solution. Finally, conventional paraffin sections and hematoxylin eosin (HE) staining were used to observe the morphological changes of gastric mucosa tissue.

### RNA sequencing and analysis

After total RNA was extracted from the sample, mRNA was enriched with magnetic beads with Oligo (dT). The mRNA fragments were divided into fragments by fragmentation buffer. The first cDNA strand was synthesized by six-base random primers using the fragment mRNA as template. The second strand of cDNA was synthesized by adding buffer solution, dNTPs, RNase H and DNA polymerase I, purified by QiaQuick PCR kit and eluted by EB buffer solution. After terminal repair, base A and sequencing joint were added, the target fragment was recovered by agarose gel electrophoresis, and then PCR amplification was performed to complete the preparation of the whole library. The constructed library was sequenced by Illumina HiSeqTM.

The reads comparison tool bowtie2(2.2.8)^32^ was used to compare High Quality Clean Reads to the ribosomes of *Tupaia belangeri*, and the reads of ribosomal RNA were removed. The retained data were compared to the reference genome (https://www.ncbi.nlm.nih.gov/genome/?term=Tupaia+chinensis) of *Tupaia belangeri* using comparison software Tophat2(2.1.1)^33^. Then, we applied Expectation–Maximization algorithm for Allele Specific Expression (EMASE)^34^ to quantify gene expression from the individual aligned RNA-seq data. Count data were normalized using DESeq2^35^ variance stabilizing transformation. Tissue levels of mercury and physiological parameters were statistically evaluated using GraphPad Prism® 7.05 using one-way analysis of variance (ANOVA) followed by Tukey's multiple comparison test. Qlucore Omics Explorer 3.5 was used for statistical analysis of transcriptomic data. Prior to statistical analysis, pre-processed RNA seq data were log2 transformed. Data were analyzed using ANOVA comparing the two groups followed by planned contrasts, to investigate the specific comparisons between groups. In the omics analyses, *p*-values were used at a threshold of *p* < 0.05 for statistical significance. The data were further examined by principal component analysis (PCA). Estimation abundances of member immune cell types related to immune infiltration using CIBERSORTx (https://cibersortx.stanford.edu/) based on gene expression data.

The differentially expressed genes were analyzed for enrichment using the Gene Ontology (GO) database and the KEGG pathway^36^. The known and predicted protein–protein interactions (PPIs) from STRING database version 11.0, http://www.string-db.org/) were downloaded. The interactions included direct (physical) and indirect (functional), derived from experimental repositories and computational prediction methods. The subnetwork was identified with highest degree by MCODE in Cytoscape v3.9.1^37^. In the PPI network, each edge denotes a score to quantify the interaction confidence, that is, the likelihood of an interaction.

### Proteomic analysis

Protein was extracted from tissue samples consistent with mRNA extraction. The protein concentration was determined by BCA protein quantitative kit. DTT and iodoacetamide were used to break the disulfide bond and reductively alkalize the protein, so as to fully enzymolize the protein later. Enzymatic hydrolysis of proteins using trypsin. The iTRAQ/TMT technique was used for proteome quantification. Mix the labeled peptide segments in equal amounts and perform reverse pre separation at high PH. Perform low pH nano-HPLC–MS/MS (Orbitrap Fusion) liquid chromatography-mass spectrometry analysis on the pre separated components, with data dependent acquisition (DDA) as the data acquisition mode.

After mass spectrometry, the raw data were extracted, analyzed, and removed isotopes by Mascot Distiller version 2.6^38^, and then converted into Mascot Generic Format (MGF). We perform database searches of MS/MS maps using Mascot (v2.3.2)^38^. The MGF file was submitted to Mascot, and the established database was selected for database search. The mass spectrometry proteomics data have been deposited to the ProteomeXchange Consortium via the PRIDE partner repository with the dataset identifier PXD047458.

Proteins were annotated using three databases (GO, KEGG, KOG).

### Statistical analyses and bioinformatics

When the difference multiple reaches 1.2 times or more and the *p*-value value is less than 0.05 in the t-test, it is considered a differential protein. The differential proteins were analyzed for enrichment using the Gene Ontology (GO) database and the KEGG pathway.

### The real-time polymerase chain reaction (RT-PCR) for candidate genes

Total RNA from fresh tissues was isolated using the Trizol reagent (Invitrogen) according to instructions from the manufacturer, and the mRNA levels of 4 genes (*RPL4*, *ANXA1*, *GAST*, *DDC*) were evaluated by RT-PCR. First, 2 μg of total RNA was reverse transcribed to cDNA using M-MLV Reverse Transcriptase (Promega, Madison, WI, USA) according to the manufacturer’s protocol. RT-PCR analysis was performed on an ABI Prism 7500 Sequence Detection System (Applied Biosystems; Thermo Fisher Scientific, Waltham, MA, USA). Relative expression levels were calculated as 2^−△△Ct^, in which Ct represents the threshold cycle for each transcript. The oligonucleotide primers are listed as follows: *RPL4* (forward, 5′-CCGCAGAGTGAATACTACCCAA-3′; reverse, 5′-TTTCCGCCAAGTGCCATACA-3′); *ANXA1* (forward, 5′-TCAAAGCAGCATACCTCCAAGA-3′; reverse, 5′-GTCTTCATCTGTTCCAAGTCCCT-3′); *GAST* (forward, 5′-ATCCTTGCTCTGGCTCTGG-3′; reverse, 5′-CCGAAGTCCATCCATCCGT-3′); *DDC* (forward, 5′-ACAGACCTAACGGGAGCCTT-3′; reverse, 5′-CTAAAGCACACCAGCCCCAG-3′).

### Ethics statement

This study is reported in accordance with ARRIVE guidelines.

### Supplementary Information

Supplementary Figure 1.

Supplementary Figure 2.

Supplementary Tables.

## Data Availability

The sequencing data have been deposited in the NCBI Sequence Read Archive (SRA) database under the accession code PRJNA1046675. SRA records will be accessible with the following link after the indicated release date: https://www.ncbi.nlm.nih.gov/sra/PRJNA1046675. The mass spectrometry proteomics data have been deposited to the ProteomeXchange Consortium via the PRIDE partner repository with the dataset identifier PXD047458. Reviewer account details: Username: reviewer_pxd047458@ebi.ac.uk Password: pR6HLwmi. The rest of the data included in the manuscript will be available from the corresponding author on reasonable request.
