# Supplementary figures and images for: RNA sequencing and proteomic profiling reveal alterations by MPTP in chronic stomach mucosal injury in tree shrew Chinese (Tupaia belangeri chinensis)
Source: Sci Rep. 2024 Jan 2;14:74. doi: 10.1038/s41598-023-50820-y (PMC10761816; doi:10.1038/s41598-023-50820-y)

A

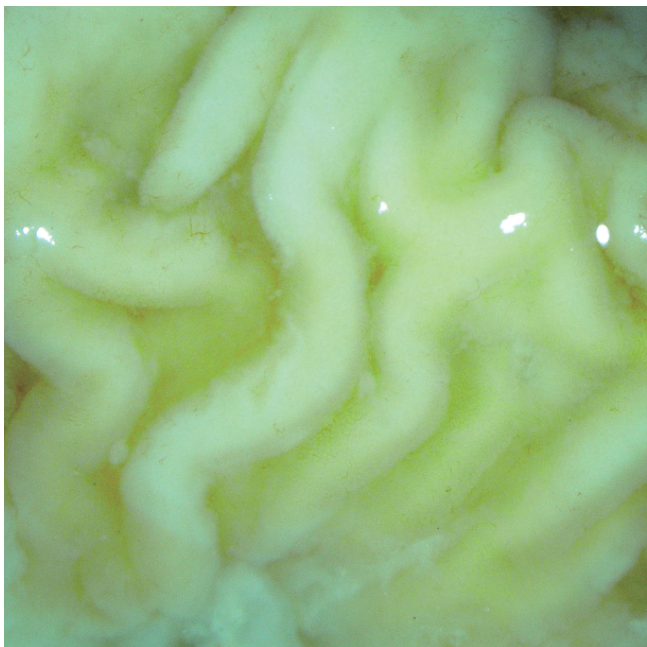

B

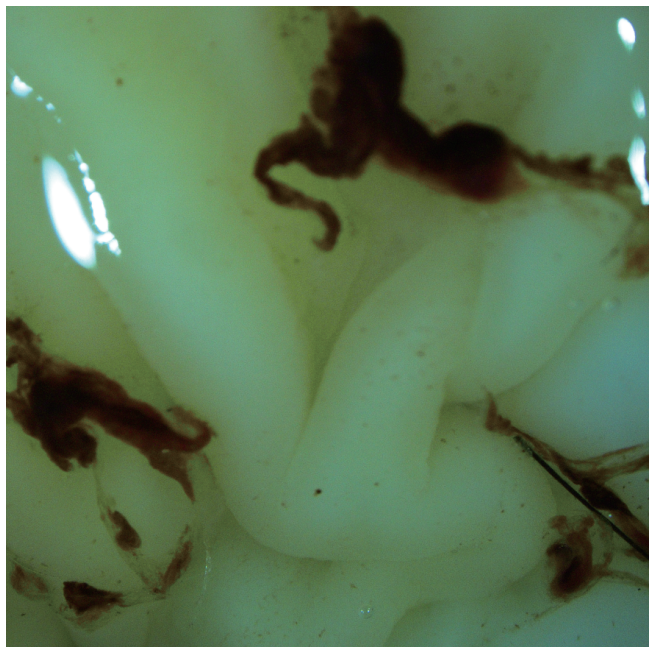

C

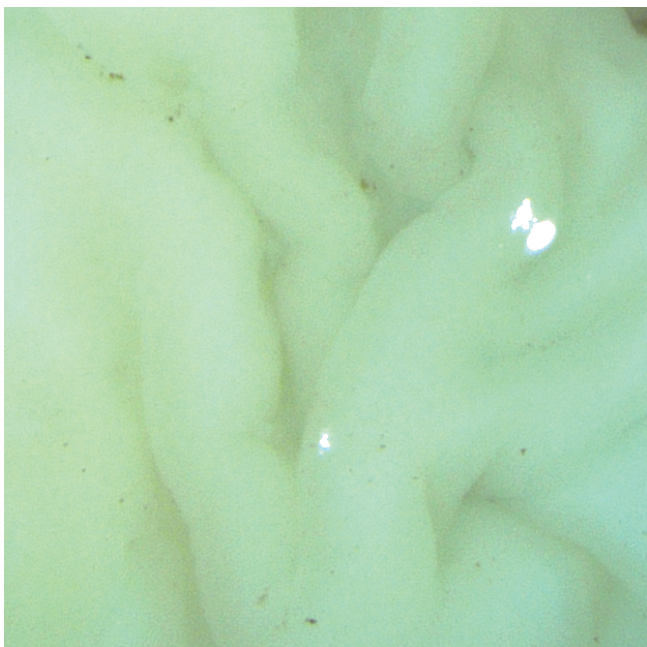

D

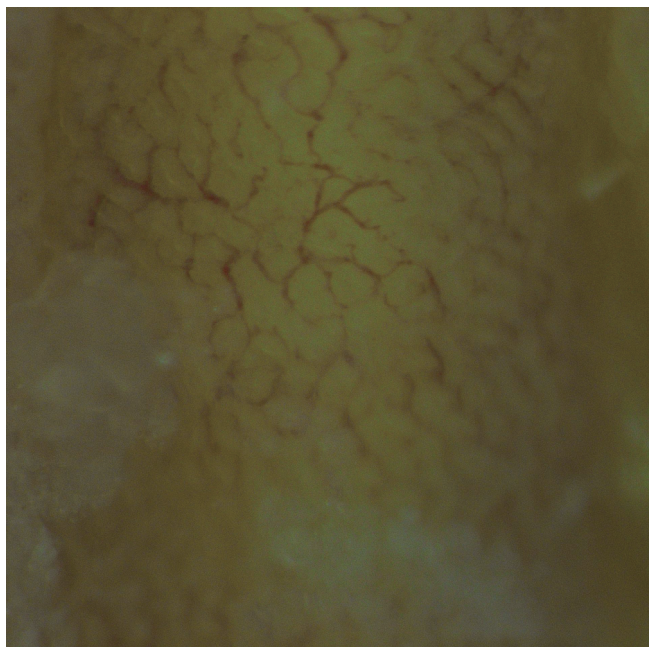

E

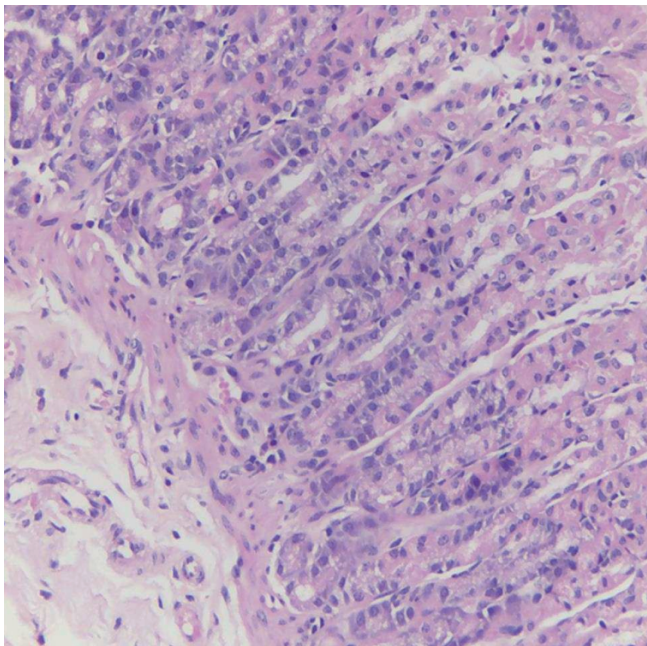

F

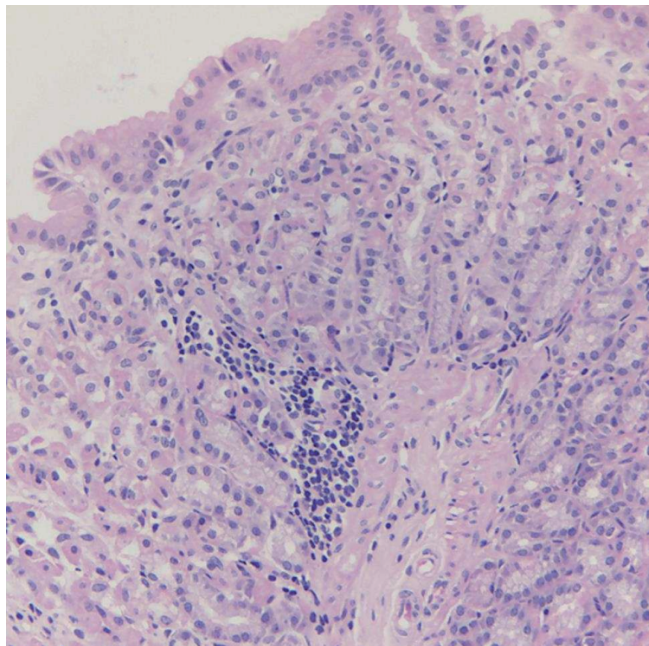

Supplement: Supplementary file 1 — Supplementary Figure 1. [file 41598_2023_50820_MOESM1_ESM.pdf]

mRNA

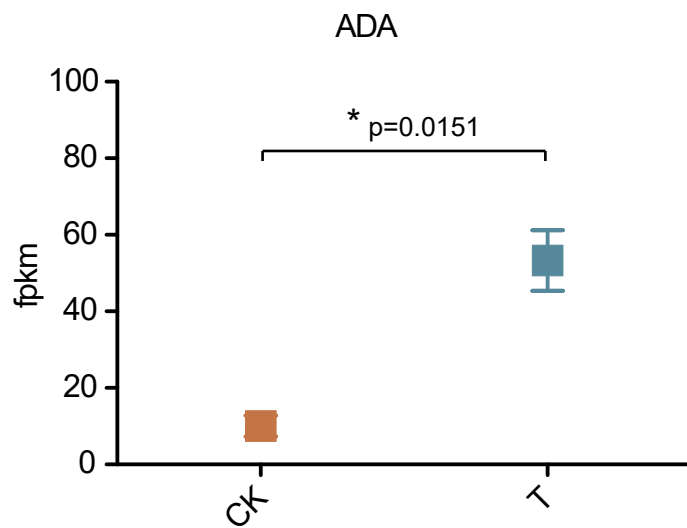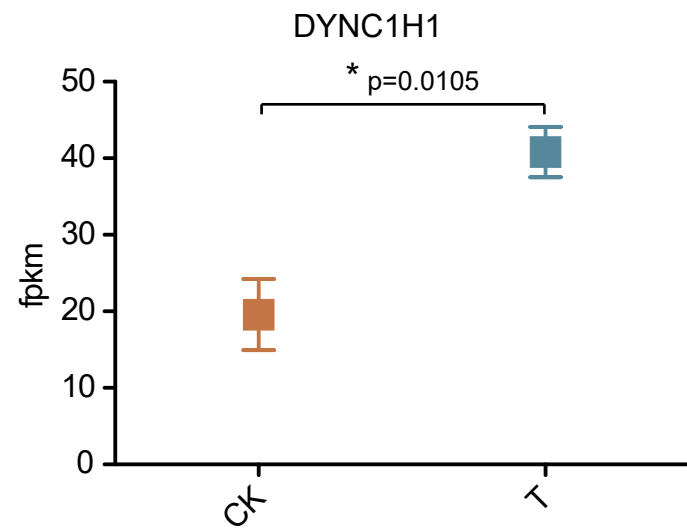

Protein

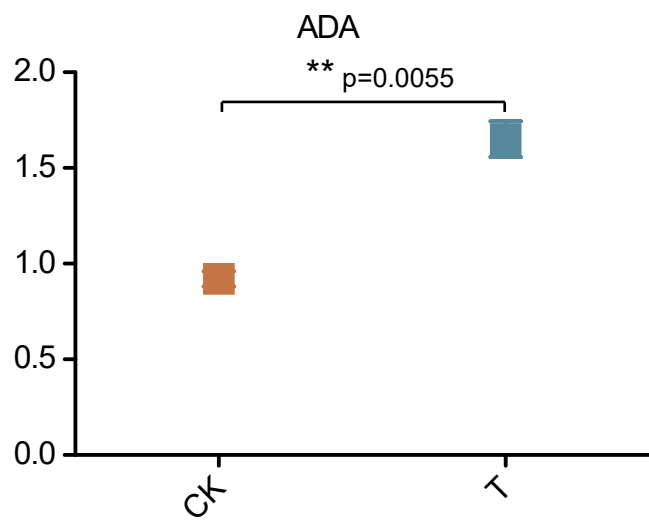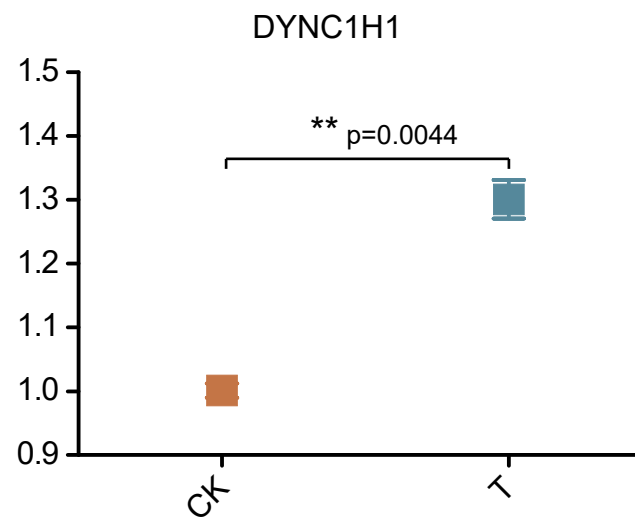

Supplement: Supplementary file 2 — Supplementary Figure 2. [file 41598_2023_50820_MOESM2_ESM.pdf]
