# Supplementary material for: RNA sequencing and proteomic profiling reveal alterations by MPTP in chronic stomach mucosal injury in tree shrew Chinese (Tupaia belangeri chinensis)
Source: Sci Rep. 2024 Jan 2;14:74. doi: 10.1038/s41598-023-50820-y (PMC10761816; doi:10.1038/s41598-023-50820-y)
Supplement: Supplementary file 3 — Supplementary Tables. [file 41598_2023_50820_MOESM3_ESM.docx]

**Supplementary Table 1: The GO enrichment of differentially expressed RNAs.**

| **Category** | **Enriched Terms** | **Genes Up** | **Genes Down** |
| --- | --- | --- | --- |
| Biological Process | metabolic process  biological phase  localization  immune system process  detoxification  cellular component organization or biogenesis  multi-organism process  rhythmic process  cellular process  cell killing  behavior  reproductive process  locomotion  reproduction  growth  biological adhesion  developmental process  biological regulation  single-organism process  response to stimulus  signaling  multicellular organismal process | 234  1  170  49  0  78  51  11  348  0  22  28  21  28  6  27  105  285  380  176  96  143 | 1496  64  711  260  8  788  301  27  2086  3  58  128  132  129  43  101  624  1614  1816  892  498  645 |
| Cellular Components | organelle  cell  cell part  organelle part  membrane-enclosed lumen  macromolecular complex  virion  virion part  nucleoid  extracellular region part  extracellular region  other organism  other organism part  cell junction  synapse  supramolecular fiber  extracellular matrix component  synapse part  extracellular matrix  membrane  membrane part | 302  311  311  124  28  63  0  0  1  63  64  0  0  14  10  1  5  6  14  268  203 | 2223  2400  2400  1224  516  849  12  12  10  144  144  2  2  132  31  16  5  24  16  977  661 |
| Molecular Function | binding  catalytic activity  electron carrier activity  nucleic acid binding transcription factor activity  antioxidant activity  transporter activity  translation regulator activity  transcription factor activity, protein binding  molecular function regulator  structural molecule activity  signal transducer activity  molecular transducer activity | 311  196  0  28  1  65  1  11  21  8  38  50 | 2142  999  4  140  9  119  4  84  136  66  82  115 |

**Supplementary Table 2: The KEGG enrichment of differentially expressed RNAs.**

| Pathway | Gene Number |
| --- | --- |
| Metabolic pathways  Fat digestion and absorption  Ribosome  Protein digestion and absorption  Pancreatic secretion  Proteasome  Oxidative phosphorylation  Starch and sucrose metabolism  Bile secretion  Mineral absorption  Ribosome biogenesis in eukaryotes  Metabolism of xenobiotics by cytochrome P450  Glycerolipid metabolism  Mismatch repair  Protein export  Pentose and glucuronate interconversions  Proximal tubule bicarbonate reclamation  Spliceosome  RNA transport  Purine metabolism  Two-component system  Ascorbate and aldarate metabolism  Carbohydrate digestion and absorption  Lipoic acid metabolism  DNA replication  Nicotinate and nicotinamide metabolism  Nucleotide excision repair  Base excision repair  Biosynthesis of secondary metabolites  Drug metabolism - other enzymes  Retinol metabolism  Linoleic acid metabolism  Drug metabolism - cytochrome P450  Porphyrin and chlorophyll metabolism  Pyruvate metabolism  Galactose metabolism  Arachidonic acid metabolism  Peroxisome  Vitamin digestion and absorption  Circadian rhythm - fly  Alanine, aspartate and glutamate metabolism  Benzoate degradation  Steroid hormone biosynthesis  Fructose and mannose metabolism  Propanoate metabolism  Phagosome  Arginine biosynthesis  Longevity regulating pathway - worm  Antigen processing and presentation  Renin-angiotensin system | 212  21  74  26  25  16  39  16  22  16  20  19  17  8  9  11  8  30  34  33  8  9  12  4  10  9  11  9  59  11  14  9  15  10  10  8  15  15  6  3  8  2  12  8  8  28  5  16  17  6 |

**Supplementary Table 3: The GO enrichment of differentially expressed proteins.**

| **Category** | **Enriched Terms** | **Genes Up** | **Genes Down** |
| --- | --- | --- | --- |
| Biological Process | immune system process  signaling  multi-organism process  multicellular organismal process  developmental process  cellular component organization or biogenesis  localization  response to stimulus  biological regulation  metabolic process  single-organism process  cellular process | 27  43  45  74  75  75  89  118  175  223  226  232 | 22  42  49  97  99  146  130  115  192  149  242  255 |
| Cellular Components | cell junction  extracellular region part  extracellular region  membrane part  membrane-enclosed lumen  macromolecular complex  membrane  organelle part  cell  cell part  organelle | 31  32  32  39  52  84  91  132  264  264  273 | 54  45  45  112  46  138  169  187  272  272  274 |
| Molecular Function | transporter activity  molecular function regulator  catalytic activity  binding | 14  29  188  250 | 23  17  72  262 |

**Supplementary Table 4: The KEGG enrichment of differentially expressed proteins.**

| Pathway | Gene Number |
| --- | --- |
| Focal adhesion  Olfactory transduction  Insulin secretion  Gastric acid secretion  Cholinergic synapse  ECM-receptor interaction  Aldosterone synthesis and secretion  Melanogenesis  PI3K-Akt signaling pathway  Oocyte meiosis  Circadian entrainment  Calcium signaling pathway  Adrenergic signaling in cardiomyocytes  Longevity regulating pathway - multiple species  Dopaminergic synapse  Long-term potentiation  Cardiac muscle contraction  Taste transduction  Oxytocin signaling pathway  Wnt signaling pathway  ErbB signaling pathway  Glutathione metabolism  GnRH signaling pathway  Glucagon signaling pathway  Vascular smooth muscle contraction  Inflammatory mediator regulation of TRP channels  Platelet activation  Regulation of actin cytoskeleton  Protein digestion and absorption  HIF-1 signaling pathway  cAMP signaling pathway  GABAergic synapse  Galactose metabolism  Tight junction  Hedgehog signaling pathway  Arginine and proline metabolism  Phenylalanine metabolism  Regulation of lipolysis in adipocyte  Longevity regulating pathway - mammal  Amino sugar and nucleotide sugar metabolism  Ovarian Steroidogenesis  Progesterone-mediated oocyte maturation  Neurotrophin signaling pathway  Ribosome  Oxidative phosphorylation  Histidine metabolism  Serotonergic synapse  Thyroid hormone synthesis  Nitrogen metabolism  cGMP - PKG signaling pathway | 43  20  21  27  25  25  21  21  35  28  23  30  34  16  29  21  19  8  30  23  14  18  22  24  27  20  28  32  13  17  23  11  13  25  9  13  6  9  12  17  8  9  14  32  25  6  16  11  4  22 |
